# Structural Evolution during Milling, Annealing, and Rapid Consolidation of Nanocrystalline Fe–10Cr–3Al Powder

**DOI:** 10.3390/ma10030272

**Published:** 2017-03-09

**Authors:** Rajiv Kumar, S. R. Bakshi, Joydip Joardar, S. Parida, V. S. Raja, R. K. Singh Raman

**Affiliations:** 1 IITB-Monash Research Academy, Indian Institute of Technology Bombay, Powai, Mumbai 400076, India; rajiv06484met@gmail.com; 2 Department of Metallurgical Engineering and Materials Science, Indian Institute of Technology Bombay, Powai, Mumbai 400076, India; paridasm@iitb.ac.in (S.P.); vsraja@iitb.ac.in (V.S.R.); 3 Department of Metallurgical and Materials Engineering, Indian Institute of Technology Madras, Chennai 600036, India; sbakshi@iitm.ac.in; 4 International Advanced Research Centre for Powder Metallurgy and New Materials, Hyderabad 500005, India; joydip@arci.res.in; 5 Department of Mechanical and Aerospace Engineering, Monash University, Melbourne, Victoria 3800, Australia; 6 Department of Chemical Engineering, Monash University, Melbourne, Victoria 3800, Australia

**Keywords:** high-energy ball milling, nanocrystalline materials, X-ray diffraction, crystallite size, grain growth, activation energy, modified Williamson-Hall method

## Abstract

Structural changes during the deformation-induced synthesis of nanocrystalline Fe–10Cr–3Al alloy powder via high-energy ball milling followed by annealing and rapid consolidation by spark plasma sintering were investigated. Reduction in crystallite size was observed during the synthesis, which was associated with the lattice expansion and rise in dislocation density, reflecting the generation of the excess grain boundary interfacial energy and the excess free volume. Subsequent annealing led to the exponential growth of the crystallites with a concomitant drop in the dislocation density. The rapid consolidation of the as-synthesized nanocrystalline alloy powder by the spark plasma sintering, on the other hand, showed only a limited grain growth due to the reduction of processing time for the consolidation by about 95% when compared to annealing at the same temperature.

## 1. Introduction

In recent years, nanocrystalline (NC) materials are being widely investigated for their superior mechanical, physical, and oxidation resistance properties when compared to their microcrystalline (MC) counterparts [1,2,3,4,5,6,7]. NC materials, such as Fe and Fe–Cr alloys, have been synthesized by several researchers [6,8,9,10,11,12,13,14,15,16,17,18,19] using different methods, such as electro-deposition [19], severe plastic deformation [13], and powder metallurgy [14,15,16,17,18]. Among these techniques, the powder metallurgy is widely used for the synthesis of the bulk NC materials [2,4,20,21,22] because it offers several advantages over other routes. Advantages include low materials loss (<5%), less processing steps, low processing cost for bulk production and near-net-shape production over other competing process [23]. Powder metallurgy processing by high-energy ball milling that has been employed for the synthesis of NC powder of Fe [2,4,24] and Fe-alloys [1,11,25,26,27] has provided the ability to economically synthesize bulk quantities of powder. During high-energy ball milling, the powder undergoes cold welding and fragmentation upon impact with the balls, as well as develops high stresses, strain-hardening, and embrittlement that assist fragmentation [28]. The fragmentation rate eventually starts to dominate over the cold welding, resulting in refinement of the powders. Moreover, the ball milling leads to a decrease in crystallite size, an expansion of lattice parameters and an increase in grain boundaries, excess free volume, and dislocations [4,21,22].

Consolidation of the ball-milled NC Fe powders into a monolithic NC solid is a challenging task due to their high hardness [29]. Besides, a considerably high volume fraction of grain boundary area of the NC powders (when compared with the MC powders) provides a large driving force for grain coarsening during the thermal processes (e.g., annealing and sintering). The large driving force for grain coarsening poses an additional challenge for the synthesis of NC powders into a monolithic NC solid. Usually, various ex-situ consolidation processes (viz., uniaxial compaction followed by sintering [7,11] using conventional and/or microwave process) and in-situ consolidation processes (viz., hot compaction, hot-isostatic pressing [30], and spark plasma sintering [31]) are used for the consolidation of MC powder. Among these consolidation techniques, spark plasma sintering (SPS) is quite promising for the synthesis of bulk NC materials [15] due to its rapid sintering. However, various parameters such as pressure, sintering temperature, holding time, and heating rate need to be optimized in order to avoid the excessive grain coarsening during the synthesis. Avoiding such grain coarsening essentially produces the fully dense bulk NC materials.

Synthesis of NC Fe and Fe–Cr alloys, and the effect of temperature on their grain growth have been well-reported [6,8,9,10,11,12]. However, the influence of the small amount of Al addition on the structural evolution during the synthesis of NC Fe–Cr–Al alloy powders by the high-energy ball milling, and the grain growth behavior of such NC alloy at elevated temperatures have not yet been reported. These understandings are of particular importance since Fe–Cr–Al alloys are well-known to exhibit an excellent oxidation resistance at high temperatures [32,33,34,35]. There is little reported on the role of nanocrystalline structure on oxidation behavior of Fe–Cr–Al alloys. However, it is well-established that NC structure reduces the required critical content of the protective oxide forming elements (viz., Al and Cr), and also enhances their oxide formation on an NC alloy [26]. Thus, it is extremely important to first develop a proper understanding of the grain growth behavior of NC Fe–Cr–Al alloys at such high temperatures that are of interest both for the synthesis needs of these NC alloys as well as their oxidation studies. Accordingly, the high-energy ball milling was chosen in this study to synthesize NC Fe–10Cr–3Al (wt %) alloy powder. This paper primarily focuses on analyzing the powder using X-ray diffraction (XRD) and transmission electron microscopy (TEM) techniques to understand the structural changes during the synthesis, annealing and consolidation steps.

## 2. Materials and Experimental Procedure

### 2.1. Materials and Methods

Elemental iron (average particle size <40 µm and 99.9% purity) and chromium (average particle size <30 µm and 99.9% purity) powders were obtained from Alfa Aesar while aluminum powder (average particle size <2 µm and 99.9% purity) was procured from Hunan Jinhao Aluminum Industrial Co. Ltd., Xiangxi, China. These powders were blended for the composition of Fe–10Cr–3Al (wt %) and ball-milled in a tungsten carbide vial with tungsten carbide balls of diameter 10 mm, with a ball-to-powder mass ratio of 10:1. In order to avoid the oxidation of powder during milling, the powder was fully submerged with toluene. The ball milling was done for 20 h using Fritsch Pulverisette P-5 planetary ball mill (FRITSCH GmbH, Markt Einersheim, Germany) and the mill was discontinued for 30 min after every hour of milling. A small amount of powder was taken out from the mill vial after 2, 5, 10, 15, and 20 h of milling to analyze its structural changes during the ball milling.

The 20 h milled Fe–10Cr–3Al alloy powder was annealed at different temperatures between 400 and 900 °C in a tubular furnace under a non-oxidizing atmosphere of forming gas (95% Ar + 5% H_2_) for 30 min. The forming gas was purged throughout the annealing process (heating, holding, and cooling). The milled powder was then consolidated by spark plasma sintering (Dr. Sinter SPS-5000 Machine, Fuji Electronic Industrial Co. Ltd., Saitama, Japan) at 900 °C with an applied pressure of 90 MPa resulting in a fully dense pellet of diameter 20 mm. The consolidation was performed in vacuum using a high-density graphite die (inner diameter: 20 mm, outer diameter: 60 mm and height: 40 mm) and punches (diameter: 20 mm and height: 20 mm). The heating rate and holding time during the spark plasma sintering were 100 °C/min and 2 min, respectively. The spark plasma sintered (SPSed) pellets were allowed to cool under vacuum inside the SPS chamber till 120 °C.

### 2.2. Characterization Techniques

The structural changes associated with the Fe–10Cr–3Al alloy powder during milling and annealing were analyzed using XRD and TEM. The XRD line profiles of the milled and annealed powders were obtained using Rigaku SmartLab system (Rigaku Corp., Tokyo, Japan) with Cu Kα radiation (λ = 0.154056 nm) with a step size of 0.01° in the 2θ range of 30°–120°. The XRD peak positions and full-width-at-half-maximum of the milled and annealed Fe–10Cr–3Al alloy powders were obtained from the multiple peak fitting of the XRD patterns using the pseudo-Voigt function [36] in HighScore Plus software (version 3.0d, PANalytical B.V., Almelo, The Netherlands) [37]. The peak broadening of the XRD pattern of the as-received Fe powder after annealing at 900 °C for 20 h was considered for estimating the instrumental broadening constituent in this study. The peak parameters of the Fe–10Cr–3Al alloy powder were used to determine the structural changes (viz., crystallite size, lattice parameter, dislocation density, excess grain boundary (GB) interfacial energy, and excess free volume) at grain boundaries using different models such as Williamson-Hall (WH) [38,39], modified Williamson-Hall (MWH) [40] and Williamson-Smallman [41]. Errors for the quantitative analysis of these structure changes were calculated by repeating the analysis for three times for the similar samples. TEM (JOEL JEM-2100F, JOEL Ltd, Tokyo, Japan) was used to validate the crystallite size of the milled and annealed Fe–10Cr–3Al alloy powders that were calculated by XRD. The XRD was also used to determine the crystallite size of the SPSed pellets. The density and hardness of the SPSed pellets were determined using Archimedes’ principle and Vickers hardness test, respectively.

## 3. Results and Discussion

### 3.1. X-ray Diffraction of Milled Powder

**Figure 1:**
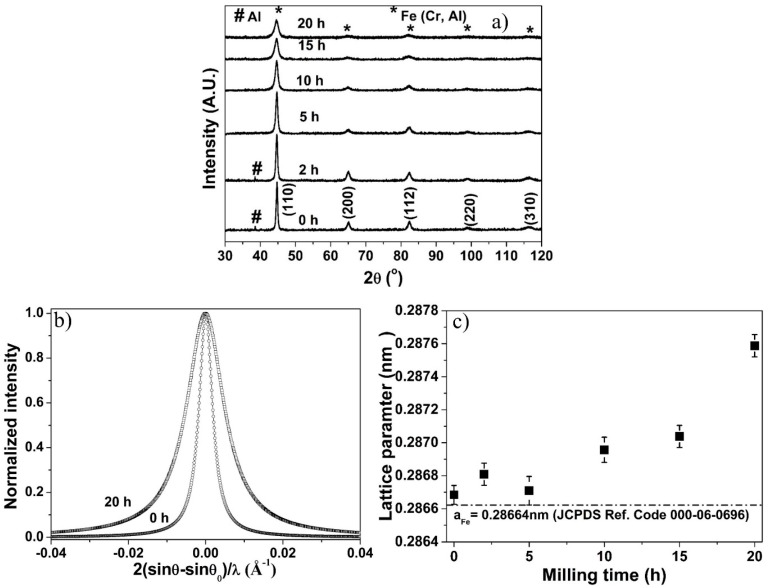
Effect of milling on (**a**) XRD patterns; (**b**) broadening of (110) peak after 0 h and 20 h of milling and (**c**) lattice parameter of Fe–10Cr–3Al alloy powder. Here, θ and θ_o_ are diffraction and exact Bragg angles, respectively.

XRD profiles of the Fe–10Cr–3Al powder blends after different milling times are shown in Figure 1a. The XRD profile of the unmilled powder (0 h) exhibits the peaks of Fe and Cr along with the prominent peak of Al. Although the prominent peak of Al is observed in the XRD profiles, Fe and Cr exhibit almost the same peak position, which is difficult to differentiate. A closer comparison of the XRD profiles suggests that the XRD peaks become broader and the peaks positions shift toward a lower diffraction angle with increasing milling time. The peak broadening of (110) peak of the 20 h milled powder is readily observed in Figure 1b. The peak broadening illustrates a reduction in the crystallite size, and the development of micro-strain during the milling of the powder, whereas the peak-shift towards a lower angle indicates the lattice expansion of the powder. Figure 1c displays the expansion in lattice parameter of the powder with increasing milling time. The lattice expansion takes place due to the formation of a solid solution of Al and Cr in Fe during milling. The formation of solid solution is suggested by the disappearance of the Al peak after 5 h of milling (Figure 1a).

### 3.2. Structural Evolution during Milling

**Figure 2:**
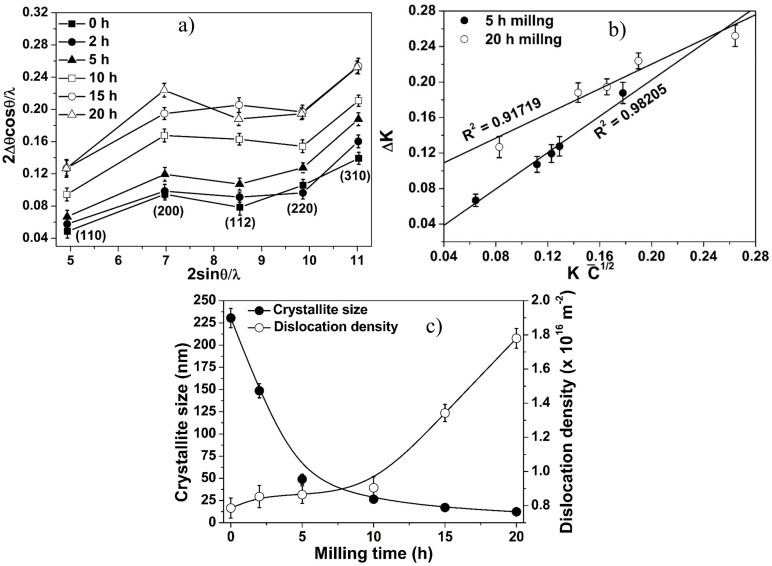
(**a**) Williamson-Hall plots; (**b**) modified Williamson-Hall plots; and (**c**) crystallite size and dislocation density as a function of milling time of Fe–10Cr–3Al alloy powder.

The peak broadening of XRD pattern is used for calculating the crystallite size of NC powders by several researchers [2,4,21,22,42,43,44] using WH and MWH techniques. Although both techniques are used extensively for calculating the crystallite size of the milled powder, the WH plot yields scattered data for an anisotropic material like Fe [2]. WH plots of the Fe–10Cr–3Al powder also (Figure 2a) exhibit scattered data points indicating an anisotropic behavior of the Fe–10Cr–3Al alloy. The extent of anisotropy of metal/alloy can be quantified by the value of an anisotropic factor, A = 2C_44_/(C_11_ − C_12_) [45]. When the anisotropic factor (A) of any metal/alloy equals 1 (i.e., A = 1), then it is taken to be isotropic (whereas, A < 1 or A > 1 indicates it to be anisotropic). Therefore, the extent of the shift in anisotropic factor from 1 is a measure of the increase in anisotropy. To calculate the anisotropic factor for an alloy, the stiffness constants (C_11_, C_12_, and C_44_) of the alloy need to be calculated. The stiffness constants for the Fe–10Cr–3Al alloy were calculated by the model proposed in the previous study [27] using the stiffness constants of Fe [46], Cr [47], and Al [46], and these constants are listed in Table 1. The anisotropic factors for Fe and Fe–10Cr–3Al alloy were calculated and found to be 2.42 and 1.67, respectively (Table 1). Although the anisotropy of Fe is reduced after the addition of Cr and Al, the additions of 10 wt % Cr and 3 wt % Al are not sufficient to change the anisotropic nature of Fe to isotropic.

The crystallite size of the milled Fe–10Cr–3Al powder cannot be calculated with sufficient accuracy using the WH method, as the WH plots of the milled powder exhibit considerably scattered data points (Figure 2a). Hence, MWH technique is preferred over WH for calculating the crystallite size [2,27]. MWH plots for the Fe–10Cr–3Al powders milled for 5 h and 20 h are shown in Figure 2b. The estimated crystallite size (Figure 2c) drops rapidly up to 5 h of milling. Subsequently, the crystallite size shows only a marginal decrease with further increase in milling time. After 20 h of milling, the average crystallite size of the milled powder is found to be ~14 nm. During high-energy ball milling, crystal defects such as dislocations are commonly introduced in the powder particles. The change in the dislocation density in the Fe–10Cr–3Al powder during milling was ascertained by the Williamson-Smallman model [41]. As evident from Figure 2c, the dislocation density shows a continuous increase with the increase of milling time.

**Figure 3:**
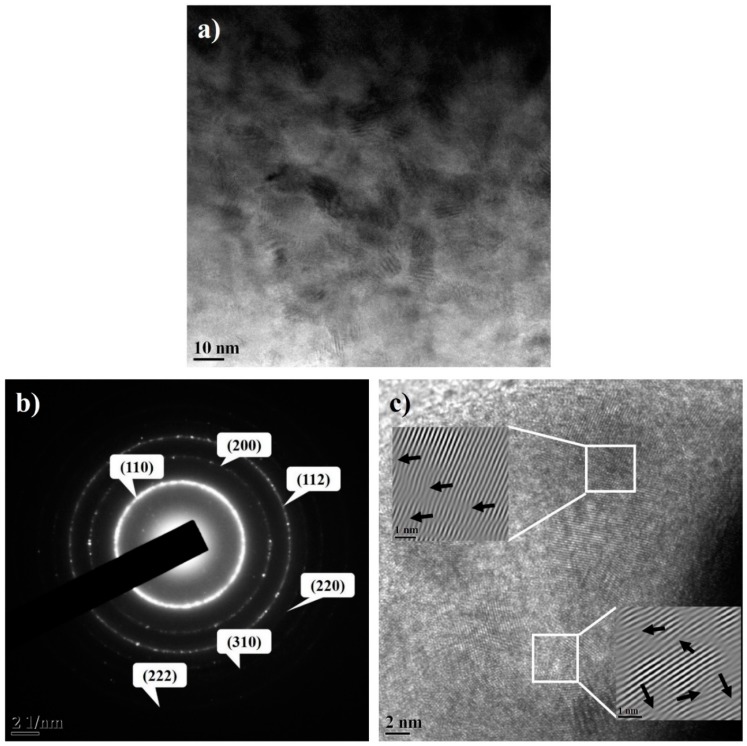
(**a**) TEM; (**b**) SAED; and (**c**) HRTEM images of 20 h milled Fe–10Cr–3Al powder. Edge dislocations are observed in the inverse fast Fourier transform (FFT) image (inset).

TEM micrograph of the 20 h milled Fe–10Cr–3Al powder (Figure 3a) suggests the formation of NC powder with the crystallite size of 15-20 nm, which is consistent with the crystallite size calculated from XRD data. Selected area electron diffraction (SAED) of the 20 h milled powder exhibits the dotted ring patterns, which also confirm the formation of the NC powder after milling (Figure 3b). TEM image of the 20 h milled powder shows more or less equiaxed grains with strong variation in contrast, indicating the presence of defects such as dislocations. The dislocations were readily observed in the high-resolution TEM (HRTEM) image (insets of Figure 3c). The presence of extra half-planes in the HRTEM image suggests the development of edge dislocations after 20 h of milling.

### 3.3. Effect of Crystallite Size on Lattice Parameter

The change in lattice parameter depends on the hydrostatic compressive and tensile stresses that establish in the materials during milling due to the interfacial stress and highly disordered grain boundaries, respectively [4,21,48]. The interfacial stress can be quantitatively calculated in term of GB interfacial energy [49]. The GB interfacial energy of the ball-milled powders is associated with the GB interfacial energy of un-milled powder and the excess GB interfacial energy [22]. The excess GB interfacial energy ($\gamma_{\mathrm{gb}}^{\mathrm{Excess}}$) can be calculated using the model proposed by Nazarov et al. [50] as follows:

(1) $$\gamma_{\mathrm{gb}}^{\mathrm{Excess}}=\frac{{\mathrm{Gb}}^{2}\rho d}{12\pi (1-\nu )}\ln\left(\frac{d}{b}\right)$$

where G is the shear modulus (79.16 GPa for Fe–10Cr–3Al), b is the Burgers vector, ρ is the dislocation density, d is the crystallite size, and ν is Poisson’s ratio. The excess GB interfacial energy varies non-monotonically with a decrease in the crystallite size during milling of pure metal and correspondingly, the interfacial stress follows a similar trend during milling [22]. However, the excess GB interfacial energy decreases monotonically with crystallite size in the case of Fe-alloy [27]. Due to the excess GB interfacial energy, the hydrostatic compression develops in the powder and leads to the lattice contraction.

**Figure 4:**
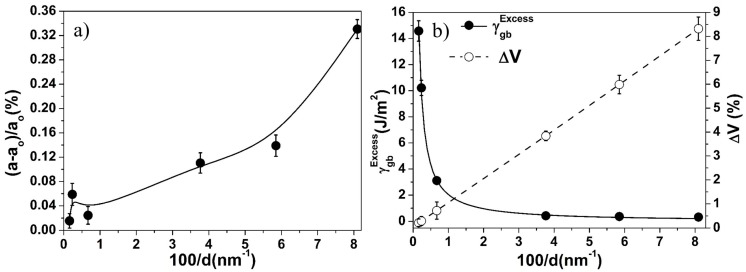
Effect of crystallite size on (**a**) change in lattice parameter and (**b**) excess GB interfacial energy ($\gamma_{\mathrm{gb}}^{\mathrm{Excess}}$) and excess free volume (ΔV) at the grain boundary of Fe–10Cr–3Al alloy powder. Here, a_o_ and a are the lattice parameter of the undistorted and distorted Fe–10Cr–3Al alloy, respectively.

The ball-milled non-equilibrium structure possesses defects such as vacancies and dislocations, which make the GB more disordered and increase the grain boundary volume called as excess free volume [51]. The excess free volume (ΔV) at grain boundaries was calculated as ΔV = ((d + ξ/2)^2^ − d^2^)/d^2^ [52], where ξ is the grain boundary thickness (~1 nm) which is assumed to be independent of crystallite size. The excess free volume induces a hydrostatic radial pressure, which leads to lattice expansion [4]. Hence, the net effect of the excess GB interfacial energy and the excess free volume influence the monotonic and non-monotonic changes of the lattice parameter of materials [21]. The change in lattice parameter of pure metals (e.g., Fe, W, Ni) varies non-monotonically with a decrease in the crystallite size [4,22]. On the contrary, the change in lattice parameter of the Fe–10Cr–3Al powder increases monotonically with the decrease in the crystallite size (Figure 4a). The monotonic lattice parameter variation indicates the dominating role of the excess free volume over the excess GB interfacial energy. The excess GB interfacial energy and excess free volume for the milled Fe–10Cr–3Al powder were calculated and shown in Figure 4b. This figure shows that the excess GB interfacial energy decreases exponentially, whereas the excess free volume increases linearly with the reciprocal of crystallite size. In addition, the excess free volume dominates over the excess GB interfacial energy in the case of Fe–10Cr–3Al powder. As a result, the lattice parameter of the powder exhibits a monotonic expansion during milling.

### 3.4. Structural Evolution during Annealing and Sintering

**Figure 5:**
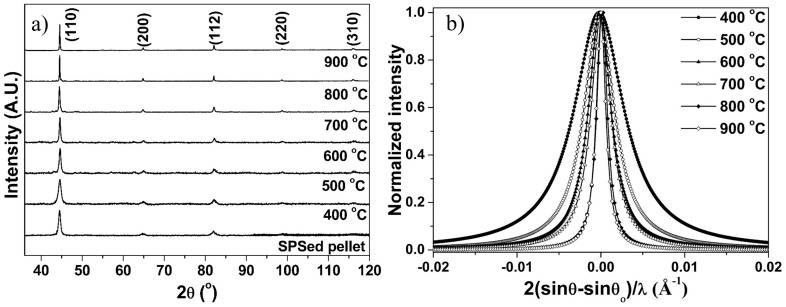
Effect of annealing temperature on (**a**) XRD line profiles of the annealed Fe–10Cr–3Al powder and the SPSed Fe–10Cr–3Al pellet; (**b**) broadening of (110) peak of the annealed Fe–10Cr–3Al powder. Here, θ and θ_o_ are diffraction and exact Bragg angles, respectively.

XRD profiles of the annealed Fe–10Cr–3Al powder and SPSed pellet are shown in Figure 5a. The peak broadening of the prominent peak of the annealed powder decreases with increasing annealing temperatures (Figure 5b), suggesting grain growth of the powder at the annealing temperatures [1].

**Figure 6:**
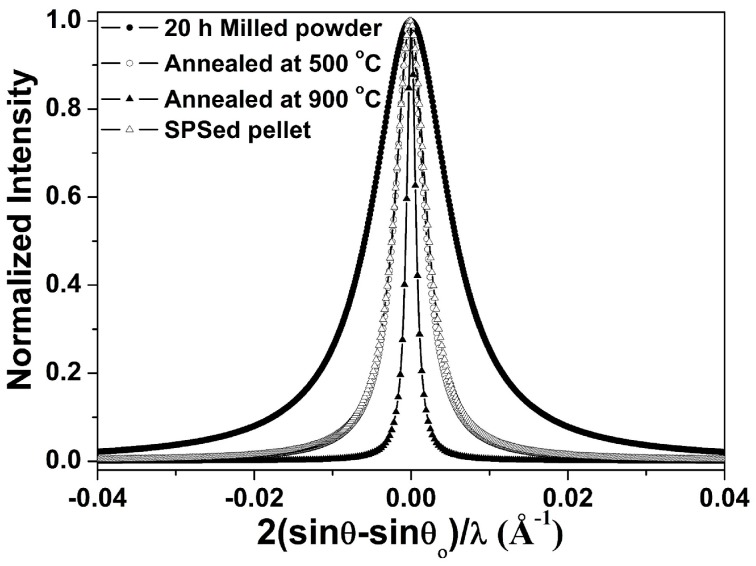
The broadening of (110) peak of Fe–10Cr–3Al alloy; 20 h milled powder, annealed powder at 500 and 900 °C and pellet sintered at 900 °C. Here, θ and θ_o_ are diffraction and exact Bragg angles, respectively.

The XRD peak broadening of the Fe–10Cr–3Al alloy (SPSed at 900 °C) is very similar to that of the powder annealed at 500 °C (Figure 6), but are remarkably less than that of the powder annealed at 900 °C. This observation suggests that the extent of grain growth in the alloy (SPSed at 900 °C) is considerably lower than that of the milled powder annealed at the same temperature. It is worth mentioning that the processing times for the annealing and consolidation of the powder at 900 °C were about 14 h (heating: 1.5 h, holding: 0.5 h, and furnace cooling till 120 °C: 12 h) and 0.5 h (heating: 9 min, holding: 2 min, and cooling till 120 °C: 20 min), respectively. Thus, the longer exposure time during the annealing may be contributing to the coarsening of crystallite size of the powder after annealing at 900 °C when compared to that after SPS at 900 °C.

**Figure 7:**
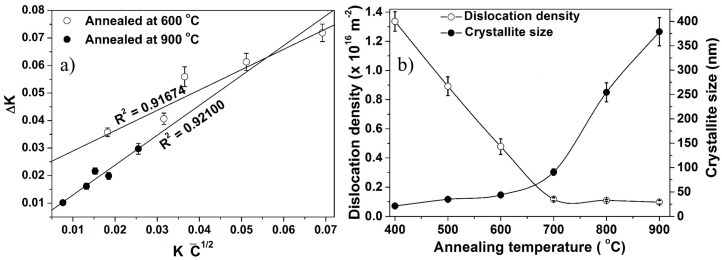
(**a**) MWH plot of Fe–10Cr–3Al alloy powder after annealing at 600 and 900 °C for 30 min and (**b**) effect of annealing temperature on crystallite size and dislocation density of Fe–10Cr–3Al alloy powder.

XRD data of the annealed Fe–10Cr–3Al powder was quantitatively analyzed for the average crystallite size and dislocation density using MWH (Figure 7a) and Williamson-Smallman methods (Figure 7b), respectively. The crystallite size increases gradually with the rise in temperature till 700 °C. The annealed powder remains nanocrystalline even after annealing at 700 °C. A further rise in temperature leads to a rapid increase in the crystallite size (Figure 7b). On the other hand, the dislocation density of the annealed powder decreases with increase in the annealing temperature (Figure 7b).

**Figure 8:**
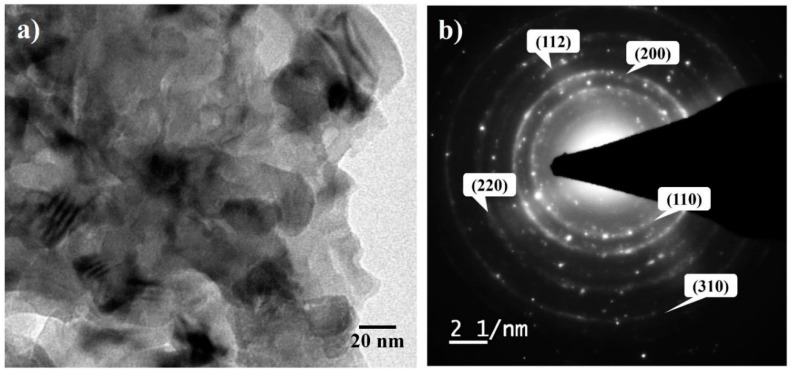
(**a**) TEM image and (**b**) SAED of Fe–10Cr–3Al alloy powder annealed at 600 °C for 30 min.

The crystallite size of the Fe–10Cr–3Al powder annealed at 600 °C was determined by TEM. TEM image (Figure 8a) illustrates that the crystallite size of the powder annealed at 600 °C is ~30–40 nm, which is consistent with the crystallite size calculated from XRD data. SAED pattern also confirms the NC structure of the powder by exhibiting the dotted ring patterns (Figure 8b). The crystallite size of the SPSed pellet (at 900 °C) is found to be ~91 nm, which is close to the crystallite size of the powder annealed at 700 °C. The density and hardness of the SPSed Fe–10Cr–3Al pellet are found to be 99% (theoretical density) and ~6.5 GPa, respectively.

### 3.5. Activation Energy for Grain Growth

It is known that the grain coarsening takes place during annealing of materials above a critical temperature. Beck et al. [53] correlated the grain size with exposure time at a particular temperature.

(2) $$D^{1/n}-D_{0}^{1/n}=\mathrm{kt}$$

where D is the instant grain size at time t, D_0_ is the initial grain size (at t = 0), n is the time exponent, and k is the temperature-dependent rate constant, which can be expressed as

(3) $${k=k}_{0}\exp\left(-\frac{Q}{\mathrm{RT}}\right)$$

where k is constant, Q is the activation energy for isothermal grain growth, R is the molar gas constant, and T is the absolute temperature. The grain size can be expressed in term of activation energy by combining Equations (2) and (3) as follows:

(4) $$\frac{D^{1/n}-D_{0}^{1/n}}{t}=k_{0}\exp\left(-\frac{Q}{\mathrm{RT}}\right)$$

To determine the activation energy for grain growth, the time exponent needs to be calculated. The time exponent for NC Fe has been reported between 0.06 and 0.35 for annealing at different temperatures <700 °C [6,8]. However, a somewhat lower time exponent (~0.08) has been reported for NC Fe–10Cr alloy, suggesting a slow grain growth [11]. The time exponent increases with the annealing temperature [6,11,53]. In addition, the time exponent can be influenced by the residual strain and grain boundary energy and presence of second phase particles in an alloy [6,54]. Consequently, Rajan et al. [54] observed further reduction in the time exponent after the addition of Mo, Y_2_O_3_, and Ti to a Fe–Cr alloy of crystallite size <20 nm, and the final value of the time exponent was found to be 0.042. These observations suggest that time exponent decreases upon the addition of thermally stable elements that can pin grain growth.

**Figure 9:**
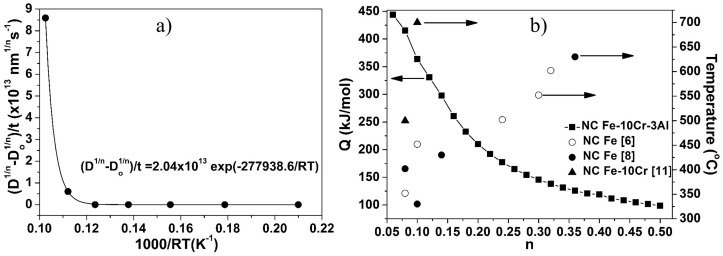
(**a**) Plot of Equation (4) for time exponent of 0.15 and (**b**) effect of temperature on time exponent and effect of time exponent on the activation energy for isothermal grain growth (arrows indicate the axis corresponds to that value).

The activation energy for grain growth of the Fe–10Cr–3Al alloy powder was calculated by plotting the left-hand side of Equation (4) versus 1/RT, which is shown in Figure 9a (only for *n* = 0.15). Similarly, the activation energy for the Fe–10Cr–3Al alloy was calculated for different values of the time exponent (0.06 ≤ *n* ≤ 0.5) after annealing for 30 min between 400 and 900 °C (Figure 9b). The activation energy decreases gradually on increasing the time exponent. Considering the time exponents reported for binary (Fe–10Cr) and ternary (Fe–9Cr–1Mo) alloys [54], the lowest time exponent of NC Fe–10Cr–3Al alloy can be estimated to be ≤0.08. Taking into account for the slow thermal grain growth of the NC Fe–10Cr–3Al alloy until 700 °C (Figure 7b) and the low time exponent (0.08), the activation energy of the alloy is calculated to be 415.3 kJ/mol. However, the highest time exponent reported for NC Fe is 0.36 and the corresponding activation energy for the NC Fe–10Cr–3Al alloy is calculated to be 125.6 kJ/mol. The low activation energy suggests higher grain growth of the NC Fe–10Cr–3Al alloy during annealing at 800 and 900 °C (Figure 7b).

## 4. Conclusions

Nanocrystalline Fe–10Cr–3Al alloy powder of crystallite size ~14 nm was successfully synthesized using high-energy ball milling. The dislocation density of the milled powder increases on increasing the milling time. Furthermore, the lattice parameter of the milled powder monotonically increases with the decrease in the crystallite size. The annealing of the milled powder at temperatures between 400 and 900 °C for 30 min causes the exponential growth of crystallite size and the reduction of dislocation density. The activation energies of the Fe–10Cr–3Al alloy for the grain growth, which is profoundly governed by the annealing temperatures, was found to lie in the range of 415.3 and 125.6 kJ/mol. A high activation energy for grain growth until 700 °C amounts to retardation of grain growth, i.e., retention of the NC structure of the annealed powder. Although a rapid grain growth is observed during the annealing at 900 °C, the grain growth is insignificant during spark plasma sintering of the milled powder at 900 °C due to the reduction of processing time by about 95%. Consequently, the spark plasma sintering of the NC powder results in a bulk NC Fe–10Cr–3Al alloy of 99.0% theoretical density.

## Figures and Tables

**Table 1:** Elastic constant, elastic modulus, and anisotropic factor for Fe and Fe–10Cr–3Al alloy.

Metal/Alloy	C_11_ (GPa)	C_12_ (GPa)	C_44_ (GPa)	Anisotropic Factor A = 2C_44_/(C_11_ − C_12_)	(hkl)	Elastic Modulus (GPa)
Fe	237	141	116	2.42	110	220.58
					200	131.81
					112	220.58
					220	220.58
					310	154.14
Fe–10Cr–3Al	222.05	112.83	91.33	1.67	110	199.69
					200	146.02
					112	199.69
					220	199.69
					310	161.66
